# The importance of having good quality indicators for care of patients with COPD: a look at hospital readmission rates

**DOI:** 10.1186/s13584-022-00528-7

**Published:** 2022-03-25

**Authors:** Michal Shani, Doron Comaneshter, Michael J. Segel

**Affiliations:** 1 Department of Family Medicine, Clalit Health Services, 56 Chen St., Central District, Rehovot, Israel; 2 Family Medicine Department, Sackler School of Medicine, Tel Aviv University, Tel Aviv, Israel; 3 Community Division, Clalit Health Services Headquarter, Tel Aviv, Israel; 4 Pulmonary Institute, Sheba Medical Center, Tel-HaShomer, Ramat Gan, Israel; 5 Division of Internal Medicine, Sackler School of Medicine, Tel Aviv University, Tel Aviv, Israel

**Keywords:** Quality indicator, COPD, Readmission

## Abstract

**Background:**

Readmission after hospitalization for acute COPD exacerbation (AE-COPD) has been proposed as a healthcare quality indicator (QI) in Israel. We studied patients hospitalized for AE-COPD, towards determining whether AE-COPD readmission is an appropriate national QI in order to improve COPD patient care.

**Methods:**

Data were retrieved for all Clalit Health Service (CHS) members age 40–90 years hospitalized in CHS hospitals during 2016 with a diagnosis of acute COPD exacerbation. Information retrieved included demographics, medical history, Charleson comorbidity score, readmissions within 90 days, chronic medication use and family physician and pulmonologist visits. Patients readmitted within 90 days were compared to those who were not readmitted. Patients were also analyzed according to whether they were hospitalized during the year before the index hospitalization.

**Results:**

In 2016 there were 70,601 members with a recorded diagnosis of COPD in CHS. Of these, 1,203 patients (1.7%) were hospitalized in a CHS hospital with a diagnosis of acute COPD exacerbation during 2016. Average age was 70.6 years, 63% were men. 78% were active smokers. 61% of the patients were readmitted to internal medicine wards within 90 days of the index hospitalization. Patients who were readmitted were more likely to have been hospitalized during the year before the index hospitalization (Odds ratio (OR) 2.5, Confidence Interval ((CI)(1.85, 3.38)) and had a higher Charlson comorbidity score (OR 1.07 (CI 1.01, 1.11)). Healthcare utilization by patients who were readmitted, both before and after admission, was generally greater. One yr mortality was 15.1% and 9.2% in those readmitted and not readmitted, respectively (*p* = 0.003).

**Conclusions:**

Readmitted COPD patients appear to be the sickest group of COPD patients with advanced disease and poor prognosis, and it may not be possible to prevent readmissions. This questions the utility of COPD readmissions as a healthcare quality indicator.

## Background

Quality indicators (QI) are widely used to improve healthcare quality [1]. Selection of a good QI is a complex process. The major criteria for a useful QI are importance of the subject, and that the putative QI is evidence based. The indicator must be measurable from good quality available data, and easy to calculate. The indicator should be clear and easy to interpret, since it should inform and influence public policy, alter behaviour of health care providers, and/or increase general understanding by the public in order to improve quality of care and population health [2].

COPD is a chronic lung disease characterized by airflow limitation. It is the third leading cause of death in the US [3]. The Global Burden of Disease Study 2015 estimated the global prevalence of COPD at about 174 million cases [4]. In an Israeli study, 22% of cigarette smokers with a history of at least 20 pack-years had COPD [5]. As a prevalent disease COPD carries a huge economic burden. It was estimated to result in direct costs of $29.5 billion and indirect costs of $20.4 billion in 2010 in the US [6]. Hospitalizations are the largest cost category for COPD patients, accounting for 38–93% of exacerbation-related cost [7]. Thus COPD admissions represent an important issue in healthcare, of great interest to both patients and heathcare providers.

The Israeli QI Program for hospitals was established by the Ministry of Health in 2013. New QIs are added to the program annually, after a formal assessment process. Hospital readmissions of Chronic Obstructive Pulmonary Disease (COPD) patients were assessed as a potential QI in this program. The suggested QI was hospital readmissions of COPD patients within 90 days of the index hospitalization. The QI Program sought an indicator influenced by both hospital care and community care. When the time-frame for readmission is extended the influence of the community care increases. The proposed 90-day time-frame was considered to strike a good balance between hospital and community care.

Readmissions can be easily recognized from computerized electronic medical record data and therefore calculation of readmission rates is feasible and easy to interpret. However it is not clear whether the AE-COPD readmission rate is likely to respond to improved patient care.

## Aim

Using data from a large HMO in Israel, we studied patients with COPD hospitalized for an acute exacerbation, towards determining whether AE-COPD readmission is an appropriate National QI. Specifically we sought to determine whether the readmission rate is likely to be improved by improving quality of care in COPD.

## Methods

This was a retrospective cohort study. Israel has national health insurance with universal access to medical care. Health care is delivered by 4 health maintenance organizations (HMOs). Clalit Health Services (CHS) is the largest HMO in Israel, serving 52% of the population. In addition to community health services for members, CHS has 8 general hospitals throughout the country. All the hospitals are Joint Commission International accredited and are under tight quality control of both CHS headquarters and the Ministry of Health.

Patient records in CHS have been completely computerized for two decades and an extensive healthcare database has been created. Patient records include demographic data, working diagnoses, medications, lab results, hospitalizations, referrals, and administrative data. Demographic data are updated directly from the population registry of the Ministry of Interior.

Data was retrieved for all CHS members age 40–90 years. All members who were hospitalized during 2016 in a Clalit-owned general hospital with a main diagnosis of AECOPD were included. The index hospitalization was the 1st hospitalization with a main diagnosis of AECOPD during 2016. Patients who died during the index hospitalization or who were readmitted to an oncology department in the next hospital admission were excluded.

Data were extracted from both community and hospital records. Information retrieved included demographic information, medical history, Charlson comorbidity score, readmissions within 90 days to internal medicine or pulmonology departments, relevant medications purchased in the community from 6 month before and until 3 months after the index hospitalization, family physician visits during the year before and the 3 months after index admission, and pulmonologist visits during the year before and the year after the index hospitalization. Patients with low socioeconomic status were defined as those exempt from healthcare payments, based on their income, by the National Insurance Institute (Israel’s Social Security Agency).

Cases were divided into those who were readmitted within 3 months and those who were not. We further analyzed cases according to whether they were hospitalized in a medical or pulmonary ward during the year before the index hospitalization.

### Statistical analysis

We compared demographic and clinical characteristics of patients who were readmitted within 90 days to those who were not readmitted. The Chi-square test was used for categorical variable and t-test for continuous variables. Multivariate logistic regression analysis was used to determinate factors associated with readmission.

STATA 8.0 statistical software (Stata Corp. College Station, TX, USA) was used for statistical analysis.

The study was approved by the local ethical committee at Meir Medical Center, Kfar Saba, Israel.

## Results

**Fig. 1:**
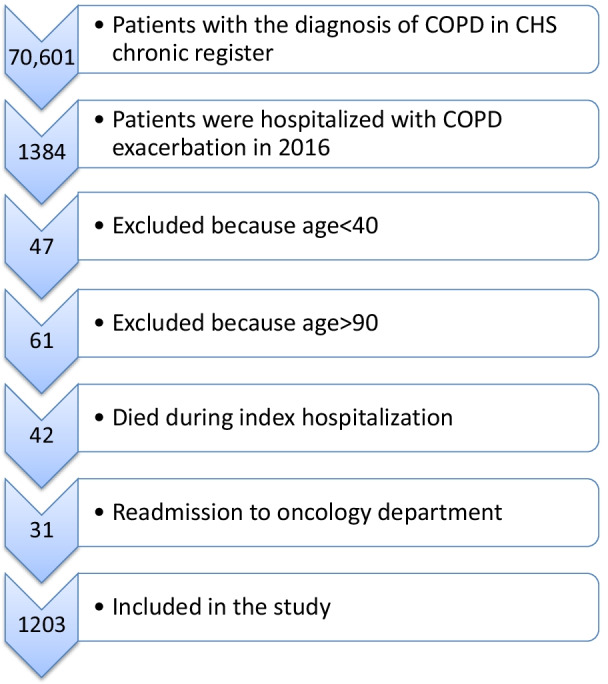
Study flow chart

We identified 70,601 CHS members who had a recorded diagnosis of COPD in 2016. Of these, 1,203 (1.7%) were hospitalized in a CHS hospital with a diagnosis of AECOPD during 2016 (Fig. 1), and were included in this study. The average age was 70.6 ± 11.0 years, 63% were men, and 73% had performed spirometry at least once. 77% were active smokers. 61% (737) were readmitted within 90 days of the index hospitalization; 36% (430) were readmitted within 30 days. The average time to readmission was 29.6 ± 24.2 days. 80% of patients had been hospitalized in a medical or pulmonary ward during the year prior to the index admission. Patients who were readmitted within 90 days after the index admission were more likely to have been hospitalized during the year before the index hospitalization (86% vs 70%, *p* < 0.0001).

Table 1 details patient characteristics and comorbidities. Patients who were readmitted had a higher Charlson comorbidity score (4.6 ± 3.0 vs. 4.0 ± 2.8, *p* = 0.0001). Length of stay of the index hospitalization (Tabel 2) was numerically shorter in those readmitted, but did not quite reach statistical significance (4.8 ± 5.0 vs. 5.5 ± 7.3 days, *p* = 0.07).

**Table 1:** Patient characteristics

	Total group			No hospitalization the year before the index hospitalization			≥ 1 hospitalization the year before the index hospitalization		
	Without re-admission (466)	With re-admission (737)	*p*-value	Without re-admission (140)	With re-admission (99)	*p*-value	Without re-admission (326)	With readmission (638)	*p*-value
Age (years)	70.7 ± 11.5	70.6 ± 10.6	0.80	70.0 ± 12.1	70.7 ± 11.7	0.64	71.0 ± 11.1	70.6 ± 10.6	0.48
Gender (% women)	38.8%	36.3%	0.38	38.5%	37.3%	0.85	38.9%	36.2%	0.40
Low SES (%)	57.1%	59.4%	0.42	56.4%	45.4%	0.09	57.5%	61.6%	0.20
Charleson score	4.0 ± 2.8	4.6 ± 3.0	0.0001	3.1 ± 2.4	3.2 ± 2.6	0.82	4.3 ± 2.9	4.9 ± 3.0	0.007
BMI (%)	28.3 ± 7.7	28.1 ± 6.8	0.75	28.7 ± 8.4	28.2 ± 6.3	0.61	28.1 ± 7.4	28.1 ± 6.9	0.93
Current smoker (%)	77.5%	77.5%	0.99	81.4%	79.8%	0.75	75.8%	77.1%	0.64
Asthma (%)	33.7%	38.3%	0.11	27.1%	32.3%	0.39	36.5%	39.1%	0.42
s/p MI (%)	21.2%	24.8%	0.15	15.0%	13.1%	0.68	23.9%	26.6%	0.36
CHF (%)	26.4%	36.2%	0.0004	22.1%	23.2%	0.84	28.2%	38.2%	0.002
DM (%)	39.3%	47.6%	0.004	32.8%	39.3%	0.30	42.0%	48.9%	0.04
HTN (%)	67.8%	70.7%	0.29	62.8%	53.5%	0.15	69.9%	73.3%	0.26
CRF (%)	21.9%	23.8%	0.55	16.43%	13.1%	0.58	24.2%	25.1%	0.77
Osteoporosis (%)	18.0%	20.2%	0.02	13.5%	17.1%	0.44	19.9%	24.9%	0.08

Healthcare utilization by patients who were readmitted was generally greater both before and after the index admission (Table 2). Readmitted patients visited their family physician more often both during the year prior to (17.5 ± 11.7 vs. 13.2 ± 10.3 visits, *p* < 0.0001) and in the 90 days after the index hospitalization (6.2 ± 4.0 vs. 4.2 ± 3.5 visits, *p* < 0.0001). A similar pattern was present for pulmonologist visits 46% vs 33% visited a specialist in the year prior (*p* < 0.0001) and 56% vs 32% in the year after the index hospitalization (*p* < 0.0001)).

**Table 2:** Healthcare utilization and mortality*

	Total group			No hospitalization in the year before the index hospitalization			≥ 1 hospitalization the year before index hospitalization		
	Without re-admission (n = 466)	With re-admission (n = 737)	*p*-value	Without re-admission (n = 140)	With re-admission (n = 99)	*p*-value	Without re-admission (n = 326)	With re-admission (n = 638)	*p*-value
Index hospitalization length (days)	5.5 ± 7.3	4.8 ± 5.0	0.07	5.1 ± 5.0	4.1 ± 3. 3	0.08	5.6 ± 8.0	4.9 ± 5.2	0.11
No. of family physician visits the year before index hospitalization	13.2 ± 10.3	17.5 ± 11.7	< 0.0001	8.5 ± 7.0	9.2 ± 6.9	0.44	15.2 ± 10.8	18.8 ± 11.1	< 0.0001
No. of family physician visits in 3 months after index hospitalization	4.2 ± 3.5	6.2 ± 4.0	< 0.0001	3.7 ± 2.8	5.6 ± 4.2	0.0001	4.4 ± 3.8	6.3 ± 4.0	< 0.0001
Pulmonologist visit the year before index hospitalization	32.6%	46.3%	< 0.0001	12.1%	13.1%	0.82	41.4%	51.4%	0.003
Pulmonologist visit the year after index hospitalization	32.0%	56.0%	< 0.0001	29.2%	61.6%	< 0.0001	33.1%	55.2%	< 0.0001
1 year mortality after index hospitalization	9.2%	15.1%	0.003	3.6%	12.1%	0.01	11.6%	15.5%	0.10

*Readmission within 90 days

Death within 1 year after the index hospitalization was significantly higher in patients who were readmitted (15% vs. 9%, *p* = 0.003, odds ratio (OR) = 1.7, 95% confidence interval (CI) 1.1, 2.7, adjusted for age, gender, Charlson score, socioeconomic status and hospitalization length of stay).

In order to explore the effect of frequent admissions we compared cases with at least one admission (to medical or pulmonary wards) in the year *prior* to the index admission to those who had not been admitted (Table 1). We found that the majority—964 patients (80%)—had been hospitalized in the year before the index hospitalization. Patients who were hospitalized in the year before the index hospitalization had more comorbidities and higher Charlson score (Table 1).

Those who had been hospitalized in the year prior to the index admission and required readmission after the index admission had a higher 1-year mortality rate compared to those who were not readmited after the index admission (15.5% vs 3.6%, *p* = 0.003; Table 2).

Prior admission in the year before index hospitalization was a strong predictor of both 30 day (adjusted OR = 1.9, 95% CI 1.4, 2.7) and 90 day readmission (adjusted OR = 2.5, 95% CI 1.85, 3.4) (Table 3).

**Table 3:** logistic regression model for readmission within 90 days

	Readmission within 30 days		Readmission within 90 days	
	Odds ratio	CI	Odds ratio	CI
Age	0.99	0.98, 1.00	0.99	0.98, 1.01
Gender	0.75	0.58, 0.97	0.93	0.73, 1.20
Low SES	0.76	0.59, 0.98	0.99	0.77, 1.27
Charleson score	1.05	1.01, 1.11	1.07	1.02, 1.12
Current smoker	1.34	0.97, 1.84	1.05	0.78, 1.44
Length of index hospitalization	0.99	0.97, 1.01	0.98	0.96, 1.00
Prior hospitalization	1.91	1.36, 2.66	2.50	1.85, 3.38

No significant differences in COPD readmission rates were noted between the 8 participating hospitals.

Readmitted patients were more likely to have been treated with a long-acting muscarinic antagonist (LAMA) inhaler both before and after the index hospitalization (Table 4). Overall, the percentage of patients treated with LAMA did not increase after the index admission, neither in those who were readmitted within 90 days, nor in those who were not. Notably, among those with a hospital admission in the year prior to the index admission, the percentage of patients treated with LAMA was significantly greater than that among those without a hospitalization in the prior year. Furthermore, in those who were not hospitalized in the prior year, the percentage treated with LAMA did increase significantly after the index admission (from 24 to 34%, *p* < 0.001). This increase was largely in those who were readmitted within 90 days of the index admission (from 26 to 41%, cf. 23% to 29% in those who were not readmitted within 90 days).

**Table 4:** Medication use related to index hospitalization

	Total group			No hospitalization the year before the index hospitalization			≥ 1 hospitalization the year before the index hospitalization		
	Without re-admission (n = 466)	With re-admission (n = 737)	*p*-value	Without re-admission (n = 140)	With re-admission (n = 99)	*p*-value	Without re-admission (n = 326)	With re-admission (n = 638)	*p*-value
ICS use 6 months before index hospitalization (%)	16.1%	21.1%	0.03	10.7%	10.1%	0.87	18.4%	22.9%	0.11
ICS use 3 months after index hospitalization (%)	14.1%	17.8%	0.10	8.5%	10.1%	0.69	16.6%	19.0%	0.36
LAMA use 6 months before index hospitalization (%)	34.8%	48.4%	< 0.0001	22.8%	26.3%	0.54	39.9%	51.9%	0.0004
LAMA use 3 months after index hospitalization (%)	36.9%	51.8%	< 0.0001	28.6%	41.1%	0.04	40.5%	53.4%	0.0001
SABA use 6 months before index hospitalization (%)	42.3%%	49.8%	0.01	33.5%	43.4%	0.12	46.0%	50.8%	0.03
SABA use 3 months after index hospitalization (%)	43.5%	46.4%	0.33	36.4%	41.4%	0.43	46.6%	47.1%	0.87
LABA use 6 months before index hospitalization (%)	4.9%	6.6%	0.22	2.8%	3.0%	0.93	5.8%	7.2%	0.42
LABA use 3 months after index hospitalization (%)	5.4%	8.1%	0.07	6.4%	9.1%	0.44	4.9%	8.0%	0.07

*Readmission within 90 days

*ICS* Inhaled corticosteroids; *LAMA* long-acting muscarinic antagonist, *SABA* Short-acting beta agonist

## Discussion

In this study we studied a large cohort of COPD patients hospitalized for an acute exacerbation. We found that Israeli COPD patients admitted for an acute exacerbation are highly prone to readmissions: 36% of the patients hospitalized with COPD exacerbation were readmitted within 30 days and 61% within 90 days. Mortality was considerable, and higher among these readmitted (15.1% 1 yr mortality vs. 9.2% in those who were not readmitted, *p* = 0.003). These data demonstrate that hospitalization for COPD exacerbation and readmissions after such hospitalizations are an important healthcare issue.

QI are implemented towards improving healthcare. In a study in the USA which compared readmissions between hospitals it was estimated that hospital quality contributes in part to readmission rates [8]. Although our database captured limited information regarding this issue, our findings do not support this conclusion. We did not find differences in readmission rates between participating hospitals in our study. Length of stay was 0.7 days shorter, on average, in those readmitted within 90 days, but this difference was not statistically significant, and moreover length of stay was not a significant predictor of 30 or 90 day readmission in the multiple regression model.

In contrast, the strongest predictors of readmission in our study were Charlson score and hospitalization in the year prior to the index hospitalization—an admission in the year prior to the index admission increased the risk of readmission 2.5-fold, and every additional point of the Charlson score increased the risk of readmission by 7%. The readmitted patients used more healthcare services both in the year before as well as in the 90 days after the index hospitalization—they visited their family physician more than patients who were not readmitted, were more likely to visit a pulmonologist and used more COPD medications. These data suggest that readmitted patients are “the sickest of the sick” COPD patients. Moreover the poor outcome despite increased healthcare utilization in the readmitted patients raises doubts whether the AECOPD readmission rate can be reduced by improving healthcare quality. Our data suggest a large unpreventable component in the readmission risk, suggesting that rate of readmission of COPD does not meet a basic criterion for an appropriate QI: strong evidence that improved care will lead to an improvement in the measured outcome [9]

Mortality from COPD in Israel declined by 25% between 2006 and 2016 [10] probably reflecting, at least in part, a gradual decline in smoking prevalence and better treatment for COPD. Nevertheless, our data suggest sub-optimal utilization of healthcare resources that potentially improve health status in COPD, such as specialist care in a pulmonology clinic, inhaled long-acting bronchodilators and inhaled corticosteroids. For example, in blatant contrast to practice guidelines [11], SABA use was more common than LAMA use. We did not capture data on smoking cessation interventions, but the fact that 77% of the cohort are active smokers strongly suggests there is much work to be done in this respect. Thus our data suggests there is considerable room for improvement in COPD care in Israel, particularly in the high-risk group we studied.

We propose that the primary QIs used to improve healthcare in COPD patients who were hospitalized for an acute exacerbation focus on implementation of recommended management strategies which may improve patients health status. In this patient population, these recommendations can be summarized as follows:

1. Step-up of current pharmacological therapy after each exacerbation, per GOLD guidelines [11] (i) LAMA or LABA; (ii) LAMA and LABA; (iii) LAMA and LABA and ICS; (iv) LAMA and LABA and (PDE-4 inhibitor or chronic low-dose macrolide therapy).
2. Smoking cessation intervention for active smokers
3. Enrollment in a pulmonary rehabilitation program.

Data on implementation of these steps, importantly including temporal trends, can be retrieved from administrative databases. After an initial assessment, goals can be set for healthcare providers for each component. Recording key spirometric parameters (such as post-bronchodilator FEV1 and FEV1:FVC ratio) will help insure accuracy of the diagnosis of COPD. Adding a simple score such as the COPD Assessment Test (CAT Score) or mMRC Dyspnea Score (common scores to assess COPD severity) to the electronic medical record will enable automated calculation of COPD severity according to the GOLD A-B-C-D classification, which is the basis for treatment guidelines, thus greatly improving the accuracy of monitoring of healthcare quality.

Finally, hospital admission and readmission rates, mortality and selective use of patient reported outcomes can be used to evaluate the efficacy of these QIs in achieving improved healthcare in COPD.

### Study limitations

The major limitation of this study is that the diagnoses of COPD and COPD exacerbation are based on main diagnosis at discharge. Although over 70% of the patients had performed spirometry sometime in the past, we did not capture spirometric data, and therefore we did not have confirmation of the diagnosis of COPD. We did not capture the diagnosis at readmission, but we excluded patients who were readmitted to an oncology department in order to minimize the effect of cancer patients in this study. On the other hand this is real life data which, while imperfect, represents the reality of medical care which is the basis for a variety of QIs.

## Conclusions

COPD patients admitted for an exacerbation, and more so those readmitted after hospitalization for an exacerbation, represent the sickest group of patients with advanced disease and poor prognosis, at a stage at which it may not be possible to change disease course and to prevent readmissions. Therefore readmission of COPD patients does not appear to be an appropriate QI for improving healthcare. QIs for patients who were hospitalized for AECOPD should focus on implementation of management strategies which are likely to improve patients health status, such as stepping-up of current pharmacological therapy after each exacerbation, according to the GOLD guidelines; smoking cessation interventions for active smokers; and pulmonary rehabilitation programs. Future studies can be designed to determine the effectiveness of these QIs in improving COPD healthcare.

Inter alia, our study highlights important characteristics of patients admitted for AE-COPD, contributing to a better understanding of the importance of these events.

## Data Availability

The data that support the findings of this study is available from CHS but restrictions apply to the availability of the data, which is used under license for the current study, and so is not publicly available. Data may be available from CHS upon request according to CHS policy.

## Abbreviations

QI: Quality indicator
COPD: Chronic obstructive pulmonary disease
AECOPD: Acute exacerbations of COPD
HMO: Health maintenance organizations
CHS: Clalit Health Service
LAMA: Long-acting muscarinic agonists
LABA: Long-acting beta-agonists
ICS: Inhaled corticosteroids
OR: Odds ratio
CI: Confidence interval
GOLD: The Global Initiative for Chronic Obstructive Lung Disease
